# Nitazoxanide and tizoxanide demonstrate high levels of *in vitro* activity against metronidazole-susceptible and metronidazole-resistant *Trichomonas vaginalis* clinical isolates

**DOI:** 10.1128/spectrum.02717-24

**Published:** 2025-05-22

**Authors:** Keonte J. Graves, John C. Williamson, Jan Novak, Hemant K. Tiwari, W. Evan Secor, Christina A. Muzny

**Affiliations:** 1 Division of Infectious Diseases, Department of Medicine, The University of Alabama at Birmingham 9968, Birmingham, Alabama, USA; 2 Department of Internal Medicine, Section on Infectious Diseases, Wake Forest University School of Medicine 12279, Winston-Salem, North Carolina, USA; 3 Department of Microbiology, University of Alabama at Birmingham 318277, Birmingham, Alabama, USA; 4 Department of Biostatistics, School of Public Health, University of Alabama at Birmingham 200394, Birmingham, Alabama, USA; 5 Division of Parasitic Diseases and Malaria, National Center for Emerging and Zoonotic Infectious Diseases, Centers for Disease Control and Prevention 1242, Atlanta, Georgia, USA; University of Texas Southwestern Medical Center, Dallas, Texas, USA

**Keywords:** *Trichomonas vaginalis*, drug resistance, nitazoxanide, tizoxanide, 5-nitroimidazole

## Abstract

*Trichomonas vaginalis* is the most common non-viral sexually transmitted infection worldwide. We compared the *in vitro* activity of the thiazolide nitazoxanide (NTZ) and its metabolite, tizoxanide (TIZ), with the activity of the Food and Drug Administration-approved 5-nitroimidazoles (metronidazole [MTZ], tinidazole [TDZ], and secnidazole [SEC]) against MTZ-susceptible and MTZ-resistant *Trichomonas vaginalis* clinical isolates. Frozen, stored *T. vaginalis* clinical isolates (*n* = 36) were cultured in Diamond’s trypticase-yeast-maltose media supplemented with heat-inactivated horse serum and an antibiotic cocktail. Drug-susceptibility assays for the thiazolides (NTZ and TIZ) and the 5-nitroimidazoles (MTZ, TDZ, and SEC) were performed to determine the minimum lethal concentrations (MLCs) for each *T. vaginalis* isolate and the median MLC for each drug. Of the 36 *T. vaginalis* isolates cultured, 18 were MTZ resistant and 18 were MTZ susceptible. For the 18 MTZ-resistant strains, the median MLCs for MTZ, TDZ, and SEC were 100, 25, and 50 µg/mL, respectively. By contrast, the median MLCs for NTZ and TIZ were considerably lower at 1.6 and 0.8 µg/mL, respectively. The similarity in thiazolide MLCs in all *T. vaginalis* strains, regardless of sensitivity to MTZ, suggests that NTZ and TIZ act via a different mechanism than the 5-nitroimidazoles. Future investigations will focus on the *in vivo* activity of NTZ and TIZ as well as the efficacy of thiazolides used as monotherapy or as combination therapy, particularly in *T. vaginalis*-infected patients who do not respond to 5-nitroimidazole treatment.

**IMPORTANCE:**

Investigating drug resistance and alternative treatment options for *Trichomonas vaginalis* is crucial due to the increasing prevalence of persistent infections and the potential failure of standard therapies (i.e., 5-nitroimidazoles). Trichomoniasis can lead to significant health complications, including increased susceptibility to sexually transmitted infections and adverse pregnancy outcomes. The rise of 5-nitroimidazole drug-resistant strains poses a challenge to effective treatment, necessitating ongoing research to understand the mechanisms behind this resistance. Exploring alternative treatments, such as novel pharmacological agents like nitazoxanide and tizoxanide, could provide more effective options for managing these persistent infections. Additionally, comprehensive investigations can help inform public health strategies and reduce transmission rates. Ultimately, prioritizing research in this area is essential for improving patient outcomes and safeguarding reproductive health.

## INTRODUCTION

*Trichomonas vaginalis* is the most common non-viral sexually transmitted infection worldwide (1). Metronidazole (MTZ), tinidazole (TDZ), and secnidazole (SEC) are prodrugs from the 5-nitroimidazole class and are the only Food and Drug Administration (FDA)-approved treatments for *T. vaginalis* in the United States. These three medications share a common mode of activation, passively diffusing into the hydrogenosomes of *T. vaginalis* and undergoing reduction reactions through the activity of enzymes responsible for energy metabolism (ferredoxin and malic enzyme), antioxidant defense (thioredoxin reductase), detoxification (nitroreductase), and the ferric iron reductase activity of flavin reductase 1 (2, 3). Once reduced to an active nitro radical anion, the 5-nitroimidazoles are thought to cause damage to the parasite’s DNA, deplete thiol pools, and form inhibitory adducts with proteins that disrupt different metabolic pathways, i.e., pyruvate-ferredoxin oxidoreductase (PFOR) and the hydrogenosomal malate dehydrogenase-mediated energy metabolisms (4, 5). Resistance to 5-nitroimidazoles has been documented and occurs in a subset of *T. vaginalis*-infected patients. The prevalence of drug resistance among clinical *T. vaginalis* isolates is unknown as *T. vaginalis* is not a reportable sexually transmitted infection, and drug susceptibility testing is not widely performed. However, it is estimated to be between 2% and 10% (6–9). Reliance on a single class of drugs, the 5-nitroimidazoles, for treatment presents a potential problem in the setting of persistent *T. vaginalis* infections due to drug resistance. Thus, there is an essential need for alternative *T. vaginalis* treatments using different classes of medications.

The nitrothiazolyl-salicylamide derivatives nitazoxanide (NTZ) and its metabolite, tizoxanide (TIZ), are anti-protozoal agents in the thiazolide class of drugs. Oral NTZ has been used in humans for the treatment of protozoal infections, including *Giardia duodenalis* and *Cryptosporidium parvum* (10). Activity against *Entamoeba histolytica*, as well as various helminths and anaerobic bacteria, has also been demonstrated (11, 12). Similar to the 5-nitroimidazoles, NTZ and TIZ inhibit the energy metabolism of anaerobic bacteria and protozoa, which involves the PFOR enzyme (13). Recently, a derivative of NTZ, amixicile (AMIX), was investigated for *in vitro* activity against *T. vaginalis* isolates (14). In this study, AMIX had greater activity when compared against NTZ and MTZ. However, the efficacy of NTZ, its metabolite, TIZ, and its derivative, AMIX, for use in the treatment of clinical *T. vaginalis* infections requires further study.

The goal of this study was to compare the *in vitro* activity of NTZ and its metabolite, TIZ, with the activity of the FDA-approved 5-nitroimidazoles, MTZ, TDZ, and SEC, against MTZ-susceptible (MTZ-S) and MTZ-resistant (MTZ-R) *T. vaginalis* clinical isolates.

## MATERIALS AND METHODS

This research was designated as non-human subjects’ research by the University of Alabama at Birmingham Institutional Review Board (IRB), Protocol #: IRB-300008770. Frozen, stored *T. vaginalis* clinical isolates (*n* = 36) were resuspended and cultured anaerobically in 9 mL of Diamond’s trypticase-yeast-maltose (TYM) media supplemented with heat-inactivated horse serum at 35-37°C for 24–72 hours. Once the *T. vaginalis* cultures were reestablished, 1 mL of media from the lower quarter of the culture tube was passed into 9 mL of fresh Diamonds TYM media supplemented with 110 µL of an antibiotic cocktail containing 10,000 IU/mL penicillin, 10 mg/mL streptomycin, and 25 µg/mL amphotericin B. These fresh cultures were then incubated for an additional 2–3 days before the *T. vaginalis* drug-susceptibility assay.

The *T. vaginalis* drug-susceptibility assay protocol used for the thiazolides (NTZ and TIZ) and the 5-nitroimidazoles (MTZ, TDZ, and SEC) was derived from the protocol used by the Centers for Disease Control and Prevention (CDC) (15, 16). Stock solutions of each drug were prepared in dimethyl sulfoxide (DMSO) and further diluted in Diamond’s TYM media. DMSO diluted in Diamond’s media without any drugs served as a vehicle-control solution for the assay. The drug-susceptibility assay was performed on 96-well plates with one drug tested per plate. Each isolate was tested in triplicate for each drug, allowing two *T. vaginalis* isolates to be tested at a time for each drug. The concentrations of each drug ranged from 0.2 to 400 µg/mL per plate. The plates were then incubated at 35–37°C for approximately 46–50 hours under aerobic conditions and then examined using an inverted microscope at 100× magnification to evaluate cell motility. Parasite viability was confirmed in the wells with vehicle control (the equivalent concentration of DMSO only). The lowest concentration at which no viable parasites were observed was recorded as the minimum lethal concentration (MLC) for that specific drug.

Statistical analyses were done for all drugs tested with *T. vaginalis* isolates being categorized as either MTZ-R or MTZ-S using the descriptive statistics tool in Excel in addition to SPSS Statistics 27 software (IBM, SPSS Inc., Armonk, New York). A comparison of median MLCs was done using related samples Friedman’s two-way analysis of variance to assess the *in vitro* activity of each drug.

## RESULTS AND DISCUSSION

**Fig 1:**
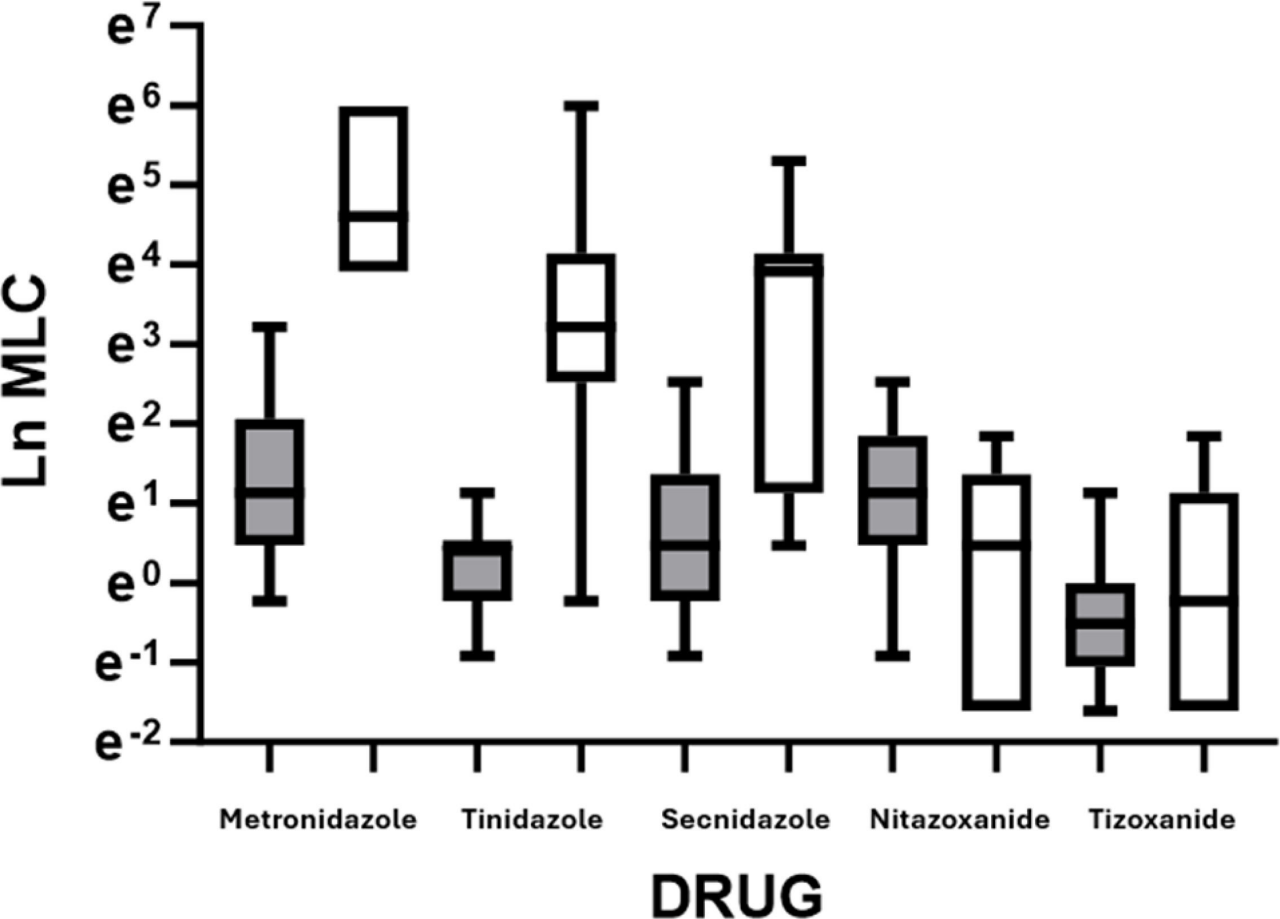
The natural log (Ln) of MLCs (µg/mL) for the different drugs between metronidazole-susceptible (gray bars) and metronidazole-resistant (white bars) isolates (*n* = 18 per group). Boxes show the median, upper 75th percentile, and lower 25th percentile of Ln MLCs for the groups. Whiskers show the upper 95th and lower 5th percentiles of Ln MLCs. Mann-Whitney comparison shows statistically significant differences (*P* < 0.0001) between susceptible and resistant isolate MLCs for the nitroimidazoles but non-significant differences (*P* > 0.05) for the thiazolides.

Of the 36 T. *vaginalis* isolates cultured, 18 were MTZ-R and 18 were MTZ-S (Table 1). For the 18 MTZ-R strains, the median MLCs for MTZ, TDZ, and SEC were 100, 25, and 50 µg/mL, respectively (Table 1). By contrast, the median MLCs for NTZ and TIZ for the MTZ-R isolates were considerably lower at 1.6 and 0.8 µg/mL (*P* < 0.001 for both). There were no observed differences in NTZ and TIZ MLCs between the MTZ-R and MTZ-S *T. vaginalis* isolates (Fig. 1), which suggests that these thiazolides act through a different mechanism than 5-nitroimidazoles. These results are consistent with prior studies that showed greater *in vitro* activity of NTZ compared to the 5-nitroimidazoles against *T. vaginalis* (13, 17, 18). The present study incorporates a greater number of well-characterized MTZ-S and MTZ-R isolates with known 5-nitroimidazole MLCs than previous studies.

**TABLE 1:** *In vitro* minimum lethal concentrations for thiazolide and 5-nitroimidazole drug susceptibility assays^*a*^

Isolate ID^*b*^		Minimum lethal concentration (µg/mL)				
		NTZ	TIZ	MTZ^*c*^	TDZ^*c*^	SEC^*c*^
MTZ-S isolates						
TV1001		0.4	0.2	12.5	1.6	3.1
TV1002		0.4	0.2	1.6	0.8	0.4
TV1007		0.4	0.2	6.3	0.8	0.8
TV1008		1.6	0.8	3.1	1.6	0.8
TV1035		6.3	3.1	3.1	3.1	0.8
TV1054		1.6	0.8	25	0.8	6.3
WF001		3.1	1.6	12.5	1.6	12.5
LUP006		3.1	0.8	6.3	1.6	12.5
LUP007		1.6	0.8	12.5	3.1	1.6
LUP008		1.6	0.4	1.6	1.6	0.8
TV1003		6.3	0.8	0.8	0.8	0.8
KTV001		3.1	0.4	1.6	1.6	0.8
KTV002		12.5	1.6	1.6	1.6	1.6
KTV003		12.5	0.4	3.1	0.4	1.6
KTV004		3.1	1.6	0.8	1.6	0.8
KTV005		3.1	0.2	3.1	0.8	3.1
KTV006		3.1	0.4	6.3	0.8	6.3
KTV007		6.3	0.4	6.3	3.1	3.1
Median MLC		3.1	0.6	3.1	1.6	1.6
MTZ-R isolates						
TV009		0.2	0.4	400	25	100
TV1062		0.4	0.8	50	0.8	25
CDC252		3.1	1.6	400	400	200
CDC0685		1.6	0.8	400	400	50
CDC1550		0.2	0.2	50	6.3	50
CDC1582		1.6	0.8	50	3.1	1.6
CDC1629		6.3	3.1	200	25	200
CDC1631		6.3	3.1	400	50	3.1
CDC1650		0.2	0.2	200	25	3.1
CDC1711		6.3	0.8	400	50	3.1
CDC5601		0.2	0.2	100	50	3.1
CDC6505		0.8	0.2	400	400	25
CDC7745		3.1	3.1	100	12.5	100
VC004		1.6	1.6	100	12.5	50
VC005		0.8	0.4	50	100	25
VC006		6.3	6.3	50	12.5	50
VC010		0.2	0.2	50	12.5	50
LUP009		1.6	3.1	100	50	50
Median MLC		1.6	0.8	100	25	50

^*a*^ Abbreviations: MTZ, metronidazole; NTZ, nitazoxanide; R, resistant; S, sensitive; SEC, secnidazole; TDZ, tinidazole; TIZ, tizoxanide.

^*b*^ TV, KTV, WF, LUP, and VC designations for sensitive and resistant *T. vaginalis* isolates from the *T. vaginalis* Repository at UAB obtained from various past and current studies conducted by the UAB STI research program. CDC designation for resistant *T. vaginalis* isolates obtained from the Centers for Disease Control and Prevention.

^*c*^ Drugs with median MLC *P* < 0.001 between MTZ-S and MTZ-R isolates using related-sample Friedman’s two-way analysis of variance.

Although NTZ consistently shows greater *in vitro* activity against MTZ-R *T. vaginalis* than 5-nitroimidazoles (MTZ, TDZ, and SEC) (13, 17), the clinical utility of NTZ for the treatment of persistent infections with 5-nitroimidazole-resistant *T. vaginalis* strains remains to be demonstrated. Oral NTZ treatment regimens were ineffective in five *T. vaginalis*-infected women detailed across three case reports/case series (19–21). Treatment regimens ranged from 1 g of oral NTZ daily for 7 days, 1 g of oral NTZ twice daily for 7 days, and 2 g of oral NTZ daily for 14 days. Treatment failure of oral NTZ is likely related to its poor absorption from the intestine. While 5-nitroimidazoles are well absorbed from the gastrointestinal tract and have good genitourinary tissue penetration, absorption of the thiazolides from the gastrointestinal tract and tissue penetration are inefficient. Upon ingestion, NTZ is readily metabolized to TIZ in the gut, which contributes to the characterization of NTZ as being poorly absorbed and having lower concentrations (22). Once NTZ is metabolized to TIZ, the plasma levels of TIZ are relatively low, compared to plasma levels of 5-nitroimidazoles (23–25). Furthermore, >99% of TIZ is bound to plasma proteins, reducing the bioavailability of TIZ to <1% (22). Despite the well-known poor absorption of NTZ, its derivative, AMIX, has been shown to exhibit higher *in vitro* activity compared to MTZ and NTZ among four *T. vaginalis* isolates (14). AMIX has also shown efficacy against *T. vaginalis* in animal models with characteristics of a systemic drug with high plasma levels, unlike NTZ (26). Future investigations should also focus on determining the efficacy of AMIX for the treatment of trichomoniasis in clinical settings.

### Limitations

A limitation of the current study involves the poor solubility of NTZ and TIZ. Neither drug is soluble in culture media; however, they have higher solubility in a few recommended organic solvents (i.e., dimethyl sulfoxide [DMSO] and dimethyl formamide [DMF]) (27, 28). Because the 5-nitroimidazole susceptibility assays are performed by first creating a stock solution in DMSO, this solvent was selected. Nevertheless, both thiazolide drugs had precipitate in the drug stock media, suggesting incomplete solubility. A proportion of the drugs not dissolving into solution signifies that some of the drugs were not available to exert their microbiologic effect. Thus, the MLC of the thiazole drugs could theoretically be even lower than what was found in this study. Additionally, differences in methodology (i.e., assay design, calculating MLC vs. half-maximal inhibitory concentration [IC_50_]) may account for variations in the MLC results between our study and past studies (13). NTZ solubility issues could also affect its clinical utility, even for intravaginal treatments. Because these drugs (NTZ, TIZ, and AMIX) are not currently in clinical use for *T. vaginalis* treatment, clinical outcome data are not available to use to define the breakpoint of susceptibility.

### Conclusion

NTZ and TIZ exhibit greater *in vitro* activity against *T. vaginalis* compared to 5-nitroimidazoles (MTZ, TDZ, and SEC), especially against MTZ-R *T. vaginalis* isolates. The similarity of NTZ and TIZ MLCs for MTZ-R and MTZ-S isolates suggests that the thiazolide mode of action differs from the mode of action of 5-nitroimidazoles. Future investigations will focus on the intravaginal activity of NTZ and TIZ, as well as the safety and efficacy of these thiazolides used as monotherapy or in combination with alternative treatment options for *T. vaginalis*-infected patients not responding to 5-nitroimidazole therapies.
